# Researches, Observations and Experiments Concerning Scorbutus

**Published:** 1847-07-01

**Authors:** 


### Sullo Scorbuto Indagini, Osservazioni ed Esperienze di *Carlo Novellis*, Dottore nelle Facultà Medico-Chirurgica.

[Annali Universali di Medicina, Vol. CXVIII. 1846.

### Researches, Observations and Experiments concerning Scorbutus. By *Charles Novellis*, M.D.

The Italian physicians seem to have greater opportunities for the observation of the phenomena of Scorbutus than fall to the lot of their brethren in this part of Europe at the present day—the comparative extinction of a disease which once committed such dreadful ravages, being one of the greatest achievements of modern hygiene. Dr. Novellis, as physician to the military prison of Alexandria, has had a great number of cases under his care, and an abstract of the interesting account of the disease he has furnished to the " Annali Universali," will we doubt not be acceptable to our readers.

The term *Scorbutus* was unknown to the ancients, and Ronseo was the first writer who employed it (in 1564), although long prior to this it was in popular use. Much difference of opinion prevails amongst authors as to whether the disease itself was known to the ancients or not, Dr. Novellis agrees with those who believe in the affirmative, and cites certain passages from Hippocrates and Paulus Ægineta in support of this view.

*Predisposing Causes.*—The principal of these are circumstances which operate changes in the organism, such as tedious convalescence, chronic disease, and cachexia, however induced. In a given number of persons, the indolent and inactive will be those most liable to suffer, and thus, in long voyages, it is upon the marines rather than upon the crew the disease commits its ravages. Hence the necessity of keeping the inmates of prisons employed. A sedentary life, confinement in close places or in the vicinity of foul air, prolonged moral affections—and in fact all that can influence the gastro-hepatic systems, in like manner predispose. If a too inactive life has this effect, so on the other hand have exhausting labours, as the forced marches of armies, &c. The cold and humidity of the climates have been cited as explanatory of the endemic prevalence of the disease in Greenland, Russia, Holland, &c.; but, if this be correct, Dr.

Novellis observes some unknown influences must, in other regions, neutralize the effects of these.

"In our Alps are districts that, for six months in the year, enjoy not the sun's rays, which are damp, and peopled by filthy peasants, who, for months together, breathe an atmosphere into which a ray of light scarcely penetrates, and who are yet unaffected by this disease. I had an opportunity of verifying the same fact in the valley of the Chison, when on duty at the fortress of Fenestrelle, near where an immense number of districts contain only damp, dirty, dark caves and huts, in which human beings are shut up with the dirtiest animals, fed with the worst aliments, deprived of all fresh articles of diet, especially vegetables—and yet is the scorbutus unknown to the inhabitants of these alpine rocks."

*Occasional Causes.*—Dr. Novellis' investigations lead him to agree with Millman, in assigning indigestible, insufficient, or innutritive food and vehement passions of the mind as the chief causes of the development of this disease; and does not believe in the supposed injuriousness of salted meats, providing these be of good quality and properly prepared. The explanation of the development of the disease during long voyages sometimes defies the ingenuity of the most attentive observers, the vessels being supplied with every appliance conducive to the health of their inmates; while other vessels in defective sanitary conditions entirely escape.

"In this point of view the citadel of Alexandria presents circumstances worthy of attention. It possesses two prisons, one civil the other military. The former is exposed to the north and is damp, dark, and badly ventilated. The latter has a north-western aspect and consists of two floors, the ground one being a little damp, but the other perfectly healthy. The civil prisoners were not subjected to any labour, but led completely idle lives, only going into the air for two hours in a kind of court. Their diet consisted of 24 ozs. of bread per diem, 4 ozs. of rice morning and evening, with 4 ozs. of fresh or 2 ozs. of dry vegetables, with butter or lard as a condiment in proportion. After two years of such seclusion, no example of scorbutus presented itself. The military prisoners are usually young and robust, are much in the open air, and actively employed. Their diet is somewhat more sparing (24 ounces in all) than the above, except when very laboriously employed, when it is somewhat increased, and a few ounces of meat and wine added. Among them scorbutus is of very frequent occurrence.

"I can only explain this difference by supposing that the civil prisoners, more advanced in years, and leading sedentary lives, are sufficiently nourished; so that a physiological condition of nutrition still being maintained, though amid so many morbigenous causes, they continued exempt from scurvy. I am the more persuaded of the correctness of this opinion, as formerly this civil prison was the receptacle for convicts condemned to hard labour, and who, living upon the poorer diet as the soldiers, were continually scourged by this disease—assimilation in them being in a vitiated condition."

Most writers upon this disease have noticed the agency of the various passions in its production. Dr. Novellis relates the case of an officer confined in a warm, dry, comfortable apartment of the fortress of Fenestrelle, having ample food with exercise in the open air for several hours, but who was firmly persuaded that at a given epoch he should be set at liberty. Disappointed of this he fell into a state of the deepest dejection, under the influence of which scorbutus soon became developed, which obstinately resisted all remedial agency, until at last the desired pardon arriving, joy again illumined his features, and his health was rapidly re-established.

The powerful effect of humidity upon the development of the disease was shown in the military prison of Alexandria, wherein our author observed thirty cases among those who slept on the ground-floor, and but one in those who slept above—the men being all placed under similar circumstances excepting as regards dormitories. He considers that the cold humidity exhaling from walls is especially pernicious, dampness uniting with heat inducing a different class of affections.

The disease has been divided into varieties according to the circumstances under which it has been observed or the nature of dominant theories. Dr. Novellis believes a natural division to be a non-febrile and a febrile variety.

*Apyretic or Chronic Scorbutus* is by far the most common affection, and perhaps no other disease presents so multiform a category of symptoms. It possesses no one pathognomic sign, several concurrent symptoms being required to characterize it. Of these, the chief are a pallor of the countenance, and an expression of melancholy which is *sui generis*—swollen, red, and bleeding gums—fetid breath—more or less deeply-coloured violaceous spots dispersed over the surface, especially of the lower extremities—pains in the hips and legs—induration or swelling of the calves, knees, or feet—amidst all which infirmities the patient retains his appetite and relish for food, and digests it. These symptoms are liable to endless varieties and anomalies, and none more so than the spots which assume different shapes and colours. Of one of these varieties Dr. Novellis remarks:—

"Few authors make mention of the *granular* eruption, cases of which are yet by no means rare. The skin of the lower extremities puts on the aspect of what is called 'goose-skin,' each little pustule having a livid brownish apex. Rarely do they enlarge, but some of them which have taken on a furuncular aspect I have opened with a lancet and found them to consist of only a subcutaneous sac filled with black blood. Sometimes they cicatrize afterwards and sometimes fill again, but they are always insensible."

The progress of the disease may be naturally divided into four stages, namely, the precursory, that of invasion, of increase, and the extreme stage. As the author's description of these in no-wise differs from that usually given we need not cite it. *Morbid anatomy,* he observes, has, as yet, thrown little light upon the nature of the affection. "The post-mortem examination of a person who has so long suffered in every form and in every system of the economy, always presents to the anatomist a mass of strange and inconstant phenomena, useless as a guide to practice." Few parts of the body indeed are there but offer traces of the diseased actions which have prevailed.

*Pathology of Scorbutus.*—The great diversity of opinions that have prevailed upon this subject sufficiently testifies to its difficulty. To cite only some of these: Hippocrates, Celsus, Willis, Riverio and a number of other writers placed the seat of the disease in the spleen; Hoffman in the liver; Murray in the pancreas; Allen and Boerhaave in the blood. Milman, Haller and others opposed this hypothesis. Lind believed the disease to consist in a relaxation of the solids with a tendency of the blood to spontaneous corruption. Milman referred its production to a diminution of the vital powers. Trotter, looking upon it in a chemical point of view, asserted the want of oxygen to be the cause of its production. Kreysig, Tommasini, Baruffi and others, observing the frequency of inflammation of portions of the venous system, resolved the disease into a phlebitis, while Versari believed it to consist in a vitiated assimilation which produced an inflamed condition, first of the venous and then of the arterial system, and ultimately of one or more of the other tissues or systems.

Many writers have characterized the disease as an *asthenic* diathesis; but this will not explain many of its phenomena, such as its frequent development under the influence of chronic phlegmasia and the abuse of alcoholic drinks—the aggravation of the symptoms by stimulating, heating remedies—their constant alleviation from the use of cooling remedies, baths, vegetable diet, &c.—and the frequent complication of scorbutus, with well-marked inflammatory diseases of the lungs, heart, liver, &c.; both diseases following the same course, and yielding to the same antiphlogistic treatment. Has scorbutus then a decidedly *inflammatory* basis? The following reasons tend to an affirmative reply. 1. Its property of changing its seat, a property belonging to phlegmasiæ. 2. The swelling of the calves and joints has all the characteristics of inflammation. 3. Pathological anatomy reveals abundant signs of the various stages of inflammatory action. 4. The eminent utility of blood-letting, as testified by almost all the principal writers upon the disease. Most of the modern Italian physicians, who have had much opportunity of observing the disease, agree in this view of its phlogistic nature; but others of them deny inflammation is other than an accidental complication, the disease itself being producible under a variety of injurious influences.

"These different opinions of great authorities sufficiently prove the difficulty of arriving at a decision upon this somewhat obscure subject. Nevertheless, in consequence of the careful investigation I have made into the cases which have come under my care, I believe the following corollaries may be drawn. 1. That this disease in no respect belongs to the asthenic diathesis. 2. That apyretic scorbutus depends upon a specific diathesis, which from accidental causes may put on either a sthenic or hyposthenic form; and in consequence I consider it neither a phlebitis or an arteritis, the blood-vessels always being only secondarily affected. From this a sufficiently easy explanation of the scorbutic pathogenesis seems to result, the affection being, as Paccinotti denominates it, a *cacotrophy*, or process of mal-assimilation. Mental passions, cold-damp, insufficient or bad aliment, and indeed every thing which can interfere with the digestive functions, necessarily lead to a vitiated hæmatosis, whence arise the debility, the yellow colour of the face, the dejection of spirits, &c., which are the earliest and most constant signs of the disease. Sanguifaction vitiated (for the blood is black and fluid, and, notwithstanding the opinion of Polli, not coagulable or very slightly so), the heart and its vessels are stimulated by a fluid foreign to their ordinary mode of sensation, and the various secretory organs become slowly irritated. In a word, every system of the animal economy undergoes change and becomes perverted in its action. The secretions are incomplete, and gradually every part of the machine tends to dissolution."

*Acute Scorbutus or Scorbutic Synocha.*—Isolated examples of this disease are of rare occurrence, but it is not unfrequently met with where scorbutic influences prevail. It may be briefly defined as *inflammatory fever, accompanied by symptoms of scorbutus.* Frequently a slight disease, at other times it is a very fatal one, the swollen gums then passing into a state of gangrene with great rapidity. The diagnosis of this disease at first is not always easy. To the early symptoms of scorbutus, such as the greenish-yellow cast of countenance, swollen state of the gums, various shaped spots distributed over the surface, we may add great heat of skin and a hard and vibrating pulse, with cephalalgia. The addition of other symptoms, such as those of gastritis, pleuritis, &c. would rather indicate an inflammatory complication supervening upon ordinary scorbutus. Whatever doubts may be entertained respecting the other form, of the sthenic nature of this there can be none. General bleeding and the application of leeches to the gums, the copious use of acidulated drinks, laxatives, and sinapisms to the soles of the feet, and abstinence, with ice, or muriatic acid gargles for the mouth, constitute efficient remedies.

As this form of scorbutus has been but little noticed by authors, we will here give a short abstract of one of the three cases furnished by Dr. Novellis.

*Case.*—" Antonio B. æt. 46, of sanguine temperament and robust constitution, and liable to inflammatory diseases, had been six months in prison, when he applied to be admitted to the hospital. He had lost his fresh colour, and from being merry and gay had become gloomy. He presented all the signs of fever, as a hard, vibratory pulse, dry rough skin, pains in the head and legs, intense thirst, heated mouth and fetid breath. He was bled at once, and again in the evening, cooling drinks and abstinence being also ordered. The disease, from the pains of the extremities, was termed a *rheumatic* synocha. Next day, no improvement being observed, the blood presenting a lardaceous crust, and his gums having become painful and bleeding at the slightest touch, two more bleedings were practised. The third day all symptoms were much alleviated, save the acute pains of the lower extremities. On examining these, to my great surprize, I found them covered with violaceous petechial spots, and, joining this symptom with the swollen state of the gums, which emitted an insupportable fætor, I could not doubt the case was an example of *scorbutic synocha.* The phlogistic symptoms re-appearing, the bleedings were repeated (in all amounting to seven), and, by the seventh day, the patient might be said to be cured, but that the petechiæ and swollen gums persisted. Purgatives and fomentation of the legs were required for several days before the skin resumed its natural moist condition; and it was not until the fourth week the patient was discharged entirely well. The following year he was admitted with exactly the same symptoms and discharged cured in two weeks."

*Complications of Scorbutus.*—Other affections may become associated with scorbutus in two modes. Either the scorbutus supervenes upon any disease, when the complication may be called *primary*, or the disease may associate itself with a pre-existing scorbutus, giving rise to a *secondary* complication. This distinction is not merely scholastic but practical. In the first case, the disease is always advanced and generally chronic, and, if it is serious, the scorbutus rapidly runs through its stages, and frequently proves fatal. In the second case, the disease may associate itself with scorbutus at any period of its progress, and it is generally an angina, bronchitis, gastro-enteritis, carditis, or pneumonia. If the patient has not become exhausted by the scorbutus, and this has not advanced beyond its second stage, it and the new disease may pursue the same course, and yield to the same antiphlogistic remedies. Nevertheless the complicating disease sometimes disappears, while the scorbutus goes through its usual course. The most fearful complication, according to Lind and Milman, is typhus, whether this be primary or secondary, sufficing, as it does, to rapidly diminish the numbers of an army, or entirely destroy the crew of a vessel. Sometimes a complication acts as a *crisis*. "I have frequently observed that, after the supervention of scorbutus, towards the end of an inflammatory disease, especially bronchitis, every appearance of this has rapidly disappeared; and, in like manner, I have seen the occurrence of an intermittent banish in two days every sign of scorbutus."

*Prognosis.*—In acute scorbutus the prognosis may usually be easily delivered; but in the apyretic form it is often very difficult, as the disease may remain stationary in its second period for months, and then proceed precipitately to an unfavourable issue. When the complication is primary, little hope of a favourable termination can be held out; and when it is secondary, the prognosis depends, during the first two stages, upon the intensity of the new malady; but when this only appears in the third or fourth stage it is very unfavourable. The complication with hepatic disease is generally fatal.

*Contagion of Scorbutus.*—This question has been a subject of great dispute among the most estimable writers; but Dr. Novellis agrees with those who utterly deny any contagious property, and, after noticing the ordinary arguments favourable to his views, he observes that *acute scorbutus* furnishes an additional one, inasmuch as, unlike variola, scarlatina, and other contagious diseases, it may be communicated to the same individual repeatedly. Since his appointment to the prison-hospital at Alexandria, he has bestowed much attention upon this question of contagion, and has never met with an instance of its propagation among the servants of the establishment, nor among others of the prisoners who constantly lived and partook of the rations of the affected. So, too, he has found in the case of the convicts, who are chained two and two, and constantly live and sleep together, that although in one of these the disease may have become developed a week prior to their separation, the other has not contracted it. The predecessor of Dr. Novellis, a believer in contagion, had taken great precaution for the separation of the scorbutic patients; but he found that the discontinuance of these, and the allowing the other patients access to the secluded portion of the building, in no-wise encreased the number of those attacked by it. Assured of the con-communicability of the disease, he yet determined to try the effects of inoculation, and conveyed into the gums, arms, and legs of two volunteer convicts, some of the blood and pus from a well-marked scorbutic case. They were retained among the scorbutic patients for several weeks, being allowed a substantial diet, but no effects whatever resulted.

*Prophylaxis.*—Experience has now amply shown that this is to be sought for, not as once thought, in drugs, but in a rational hygiene, modified according to the varying circumstances of climate, locality, &c. By the aid of this, the improvement accomplished in the condition of prisons, and ships at sea has been immense. A judicious alimentation seems to be a principal agent, the portions of flesh and vegetable being duly proportioned. Dr. Baly's observations upon the anti-scorbutic properties of the potatoe are in this point of view quoted with approbation by the author.

*Curative Measures.*—Innumerable as have been the remedies vaunted for the cure of scorbutus, not only does not any one of them deserve the title of a specific, but we cannot even say that we have any certain guide for its rational treatment. Thus it is a well-known fact, that vegetable substances are of great advantage in scorbutus, and yet the disease has been frequently known to rage on board vessels well supplied with them, and Wilson furnishes us with an account of an irruption of the disease in places where vegetables were the sole diet, and which was cured by having recourse to flesh diet.

Our first effort must be directed to the removal of the patient, where this is possible, from the locality or climate in which he is in, after which our remedial agents may act efficiently. Every effort must be made to encourage him, and remove the melancholy by which he is oppressed. The effects of hope are sometimes wonderful. Thus Frank relates that, after it had been announced to the French army that its return to France was decided upon, of 220 patients suffering from scorbutus, the greater part very severely, only 18 died, the remainder becoming rapidly well.

When a prisoner presents signs of scorbutus, Dr. Novellis follows the practice of Van Swieten, of administering a laxative. He is cautious in the use of emetics, especially of antimony, inasmuch as gastritis easily complicates scorbutus. All complications of an active kind must be promptly dealt with. Thus, gastro-enteric irritation is to be relieved by applying leeches to the hæmorrhoidal vessels; and, if there is cough, palpitation, dyspnœa, or those wandering pains termed by Sydenham scorbutic rheumatism, or darting pains in the chest, with a hard full pulse, venesection is required, and although the blood drawn is black and of little density, a persistence of the symptoms will call for a further loss. Much discrepancy of opinion prevails as to the employment of venesection in *simple apyretic scorbutus.* Many declare it is indispensable, others prohibit it, and others again employ it sparingly. Dr. Cima declared, at the Milan Congress, that when he used to bleed he lost from 5 to 10 per cent. of his patients, but that since he had ceased doing so, he scarcely lost 1 per cent. In an epidemic which occurred at Montevideo in 1844, two Italian physicians, MM. Odicini and Antonini, regarding the disease as a phlebitis, resorted to depletion and other lowering remedies, and declared that the mortality in their hospital was much less than that among the patients of the French, English, and Spanish physicians, who were treated by stimulants and tonics. Dr. Novellis' own experience leads him to believe this remedy is of slight utility, unless manifest signs of plethora be present.

*Baths* are of great utility, diaphoresis being frequently induced after the second or third, and followed soon after by disappearance of the maculæ and other bad symptoms. In France they are frequently medicated, but Dr. N. prefers the simple water. The partaking of *acid drinks* of various kinds is of great advantage, although not so exclusively a curative measure as sometimes supposed. The use of *vegetables* has been prescribed by almost all writers; but some of these of a stimulant nature, such as celery, onion, garlic, &c., when used immoderately, Dr. N. has seen several times give rise to the disease. He imitated the series of comparative experiments performed at Haslar by Lind, and came to the same conclusion with that writer, that preference was not to be specially given to any one vegetable, since plants of quite opposite qualities, as the *nasturtium aquaticum* and the *lactuca sativa*, produced just the same effects. He agrees with those writers, also, who consider plants most efficacious when eaten not only fresh but uncooked. Upon the strength of the statements of Blane, Smith, and Baly, he also tried the potatoe, but found it of little or no use, and considers that it, or any farinaceous food, is improper for the treatment of the disease, however suitable an adjunct to meat diet as a prophylactic.

Dr. Novellis speaks approvingly of the *nitrate of potass*, given with acidulated drinks, so warmly praised by some authors. The preparations of *iron*, also recommended by some moderns, have been freely tried by him, but without any successful result, and often with the effect of exciting gastric disturbance and diarrhœa. He relates a series of experiments he performed on a number of prisoners, first with preparations of iron, and then with simple doses of nitrate of potash. Under the use of the first the patients continued stationary or retrograde for months together: while by the employment of the nitrate, with the aid of fresh vegetable and good meat diet, and the moderate use of wine, they rapidly got well. We must here protest against the cool manner in which our author submits those assigned to his care to a treatment which prior experience had already convinced him was a bad one, merely, as it would seem, to be able to state in figures the exact proportion in which it is so. He had already determined that iron was useless or hurtful when he instituted a new set of experiments to show how soon nitre would cure those who had been for *several months* submitted to such injurious influences. In the name of the dignity and usefulness of our art we protest against this wanton procedure.

From the time of Huxham, who relates the case of a woman who induced scorbutus by taking *marine salt* for the cure of scrofula, this substance has been reputed as a frequent cause of the disease. Russell, Lind, Milman, and others, have protested against the doctrine, but it has become somewhat rooted in popular opinion. Our author found it very prevalent, so that the food employed in ordinary was quite insipid. He increased the quantity of salt, and soon found the number of scorbutic patients diminish. So, also, he substituted for the gargles in use one composed of one ounce of salt to four ounces of water, with the effect of rapidly improving the condition of the diseased gums; the ulcerations, flux of blood, and sphacelus, once so common, now no longer appearing. The salt, too, proved a refreshing cooling application to the heated mouth.

*Differential Diagnosis.*—The author examines this in relation to the various local and general diseases scorbutus is liable to become confounded with, such as Gingivitis, Hæmorrhage from the Gums, Aphthæ, Stomatitis, Syphilis, Purpura, Petechial Typhus, &c. &c. We need only notice the two last.

The causes capable of producing *purpura hæmorrhagica* much resemble those which induce scorbutus; and the symptoms of the two diseases are very similar. Both, too, may continue in a chronic condition for months. It is of importance to turn attention to the concomitant symptom of purpura, the hæmorrhage, which is not found in the early stages of scorbutus. The scorbutic synocha is distinguished from the purpura by a formication of the surface, the livid color of the gums and the fetid breath. Although there is much resemblance between the symptoms of *petechial typhus* and scorbutus, the skilful practitioner will easily distinguish them. The third stage of the latter has been called by some writers the *typhoid stage*, on account of the prostration into which the patient falls; but this may be easily distinguished from a true typhus, since, besides the state of the gums, the fætor of the breath, and the pains in the extremities, the scorbutic patient never loses his appetite, desiring even the most extraordinary articles of diet—while in typhus the most complete anorexia prevails.

We consider that Dr. Novellis has produced a very creditable essay upon an interesting subject, and we only wish that he and his Italian brethren would furnish us with more frequent opportunities of making their acquirements better known to the practitioners of this country.

